# Effect of an Evidence-based Inpatient Tobacco Dependence Treatment Service on 1-Year Postdischarge Health Care Costs

**DOI:** 10.1097/MLR.0000000000000979

**Published:** 2018-08-20

**Authors:** Kathleen B. Cartmell, Clara E. Dismuke, Mary Dooley, Martina Mueller, Georges J. Nahhas, Graham W. Warren, Peter Fallis, K. Michael Cummings

**Affiliations:** * College of Nursing; † Hollings Cancer Center; ‡ Center for Health Disparities; Departments of § Psychiatry & Behavioral Sciences; ∥ Radiation Oncology, Medical University of South Carolina, Charleston, SC; ¶ TelASK Technologies Inc., Ottawa, ON, Canada

**Keywords:** tobacco cessation, healthcare costs, cost benefit, secondary analysis

## Abstract

**Background:**

In 2014, the Medical University of South Carolina (MUSC) implemented a Tobacco Dependence Treatment Service (TDTS) consistent with the Joint Commission (JC) standards recommending that hospitals screen patients for smoking, provide cessation support, and follow-up contact for relapse prevention within 1 month of discharge. We previously demonstrated that patients exposed to the MUSC TDTS were approximately half as likely to be smoking one month after discharge and 23% less likely to have a 30-day hospital readmission. This paper examines whether exposure to the TDTS influenced downstream health care charges 12 months after patients were discharged from the hospital.

**Methods:**

Data from MUSC’s electronic health records, the TDTS, and statewide health care utilization datasets (eg, hospitalization, emergency department, and ambulatory surgery visits) were linked to assess how exposure to the MUSC TDTS impacted health care charges. Total health care charges were compared for patients with and without TDTS exposure. To reduce potential TDTS exposure selection bias, propensity score weighting was used to balance baseline characteristics between groups. The cost of delivering the MUSC TDTS intervention was calculated, along with cost per smoker.

**Results:**

The overall adjusted mean health care charges for smokers exposed to the TDTS were $7299 lower than for those who did not receive TDTS services (*P*=0.047). The TDTS cost per smoker was modest by comparison at $34.21 per smoker eligible for the service.

**Discussion:**

Results suggest that implementation of a TDTS consistent with JC standards for smoking cessation can be affordably implemented and yield substantial health care savings that would benefit patients, hospitals, and insurers.

Tobacco use causes 18% of all deaths in the United States^1^ and accounts for a greater proportion of US health care costs than any other single factor.^2^,^3^ Tobacco use is also an established risk factor for hospital readmission due to cardiac,^4^–^8^ pulmonary,^9^,^10^ surgical, and wound healing-related conditions.^11^–^16^ In 2012, the Joint Commission (JC) issued an optional measure set for screening and treatment of hospitalized smokers, recommending hospitals document tobacco use status of all patients, provide evidence-based cessation counseling and medication during the hospital stay, provide referral at discharge for evidence-based counseling and medication prescription, and document tobacco use status ∼30 days after discharge. Despite meta-analysis evidence that adherence to these practice guidelines increases rates of smoking cessation at 6–12 months by 37%,^17^ many hospitals do not consistently provide Tobacco Dependence Treatment Service (TDTS) as recommended by the JC.^18^ There are many reasons why hospitals have not implemented the JC standards for smoking cessation, but chief among them are concerns about the costs of implementing a TDTS without clear evidence for return on investment back to hospitals and insurers who are being asked to cover the costs of the TDTS.

Six published studies have assessed cost-effectiveness of offering smoking cessation to hospitalized patients, and all reported intervention cost-effectiveness.^19^–^24^ Findings from these studies remained robust in sensitivity analyses conducted across parameters such as variability in program costs,^21^,^24^ addition of free medication to the program,^23^ quit rates,^20^–^22^,^24^ relapse rates,^19^ nonfatal disease events,^22^ mortality,^19^,^22^,^24^ and quality of life,^19^ as well as probabilistic sensitivity analyses to account for instability of the statistical models.^19^,^23^

However, review of these studies highlights important evidence gaps. First, none of these 6 studies evaluated TDTS cost outcomes using actual health care utilization and costs incurred by the study cohort. Second, only one of the 6 studies examined the impact of a smoking cessation service among a total population of hospitalized patients.^20^ The other 5 published studies were conducted among subgroups of hospitalized patients with health problems such as heart problems, chronic obstructive pulmonary disease, and mental illness. Third, nearly all existing cost studies were based upon cohorts of patients recruited into research studies where efforts were made to optimize follow-up, thus not reflecting real world implementation conditions. Four of the 6 studies used a combination of randomized controlled trial evidence and/or modeling based upon the literature, with only 2 studies using quasi-experimental designs to evaluate cost outcomes under operational program conditions.^20^,^23^

In 2014, the Medical University of South Carolina (MUSC) implemented an automated TDTS using interactive voice response (IVR) technology and a TDTS Registry (TelASK Technologies Inc.) to operationalize JC standards for tobacco treatment. This TDTS Registry links to the hospital’s admission and discharge records to identify tobacco users, automatically refers them to hospital-based cessation services where a tobacco treatment specialist (TTS) visits the patient at bedside to assess smoking behavior, talks to the patient about the importance of smoking cessation and develops a tailored treatment plan for the patient to be executed during the hospital stay. In addition, the TDTS uses IVR technology to generate automated follow-up phone calls to smokers at 3, 14, and 30 days postdischarge to evaluate their tobacco use status and transfer them to live phone-based counselors at MUSC or at the state quitline as needed. We have previously demonstrated that patients exposed to the TDTS were less likely to be smoking one month after discharge^25^ and that exposure to the service reduced rates of 30-day unplanned hospital readmissions.^26^

The current study examines the association between exposure to the MUSC TDTS and downstream health care charges during a 1-year posthospital discharge period by addressing 2 specific research questions: (1) Do patients exposed to the TDTS have lower health care charges compared with smokers not exposed to the service? and (2) What is the cost of implementing the TDTS?

## METHODS

### Study Design

A secondary data analysis was conducted to link 3 datasets to evaluate the effect of the MUSC inpatient TDTS on subsequent 1-year health care utilization charges. We used regression models to evaluate the association between exposure to the MUSC TDTS and subsequent 1-year health care utilization charges, controlling for influential covariates. We also calculated the cost of implementing the MUSC TDTS.

### Study Setting and Population

MUSC is a large tertiary care hospital in Charleston, South Carolina with ∼30,000 annual adult hospital admissions. All current smokers 18+ admitted to the hospital were eligible for the TDTS, with the exception of patients admitted for psychiatric care, same day surgery or <24 hour observation. In addition, patients were excluded for the following logistical reasons: (1) died during hospitalization; (2) were unable to communicate due to language or medical condition; (3) not discharged back home, (4) did not provide a phone number; and (5) patients readmitted who already had an active TDTS follow-up call scheduled. The study cohort consisted of eligible current smokers admitted and discharged from MUSC between November 1, 2014 and June 31, 2015.

Patients enrolled in the TDTS, were given the option to “opt-out” of the service. The plan was for those who did not opt out of the TDTS to receive a bedside consult from a TTS, IVR-based phone support, or both. However, because of resource limitations for delivering the service and other reasons, only 53.2% (1640/3081) of eligible TDTS patient received the service (ie, consult, phone follow-up, or both) while 46.8% (1441/3081) did not receive the service. Reasons for not receiving the service included failure to provide the bedside consult because the patient was unavailable when the consult was offered (ie, not accessible when visited by the TTS or discharged before seeing the TTS), and patient failure to respond to any IVR follow-up calls made within 30 days after hospital discharge.

### Data Collection and Linkage

All current smokers with an eligible index admission between November 10, 2014 and June 31, 2015 were included for analysis. An index admission was defined as the initial event for which the patient sought care (eg, initial heart attack or hip/knee replacement procedure).^27^ Index admissions that resulted in lengths of stay (LOS) >30 days, death, psychiatric care or discharge against medical advice were excluded from analysis. Psychiatric admissions were excluded because the TDTS was not implemented for psychiatric patients until 2016. Patients with LOS>30 days were excluded because prolonged LOS may indicate a condition for which intervention to control subsequent health care utilization may be overshadowed by illness severity.

Three datasets were linked to conduct the study: (1) the MUSC electronic health record database which provided information about tobacco use status for all hospitalized patients; (2) the TDTS Registry which provided information about which hospitalized patients participated in the MUSC TDTS and level of service received; and (3) the Statewide Hospital Utilization datasets which provided information on all inpatient, ambulatory surgery, and emergency department (ED) charges in South Carolina. Data linkage was perfomed in 2 stages.^26^ First, MUSC electronic health record data was linked with the TDTS Registry. Second, the linked MUSC dataset was submitted to the SC Office of Research and Statistics (SC ORS) for linkage with the SC Healthcare Utilization datasets.

### TDTS Exposure Variables

TDTS exposure was defined in 2 ways: (1) the exposed group received either a bedside consult and/or responded to at least one IVR follow-up call versus the unexposed group who received neither a bedside consult nor responded to any of the IVR follow-up calls; and (2) level of exposure to the TDTS was further defined as high, low, and unexposed, with high exposure defined as receiving the bedside consult (regardless of whether they responded to any postdischarge IVR follow-up calls), low exposure defined as responding only to the postdischarge IVR follow-up calls, and unexposed as defined above.

### Outcome Variables

Two main outcome variables were examined: (1) health care ulitization charges for patients with and without exposure to the TDTS over a 1-year period after index admission at the MUSC hospital; and (2) the cost of implementing the TDTS.

#### Health Care Charges

One-year health care charges following an index admission were estimated for patients who did and did not receive the TDTS. These charges consisted of all inpatient, ambulatory surgery and ED charges that patients in the study cohort incurred in SC during the 1-year period after the index admission. Inpatient charges for the same type of admission can vary widely based on hospital mission (for-profit, nonprofit, etc.) and insurance status of the individual. To reduce this variability, we calculated standardized inpatient charges by Diagnosis-related Group (DRG). Standardized inpatient charges were calculated based on summing all admission charges for each DRG and dividing by the number of admissions to obtain the mean charge per DRG, which was then applied to each admission based on its assigned DRG.

Overall 1-year health care charges, consisting of overall inpatient, ambulatory surgery and ED visit charges, were then compared for adult current smokers with and without TDTS exposure. These analyses were repeated to compare cost outcomes for varying levels of the TDTS (ie, low intensity vs. no exposure; high intensity vs. no exposure; low vs. high intensity exposure to the TDTS).

#### Cost of the TDTS

The costs of the intervention included salary support for the full-time TTS at 100% effort and part-time nurse manager at 30% effort based upon published median salaries,^28^ office space and equipment prorated to the TTS and program manager’s effort on project, and costs associated with the IVR follow-up calls and TDTS Registry which involved a contract with an outside vendor (TelASK Technologies Inc.). Some costs were fixed costs associated with establishing the program (eg, IT support to set up the TDTS registry, office equipment for new staff), while other costs were recurring, such as salary costs for TDTS staff and TelASK per patient charges that were based on the estimated number of MUSC inpatients who are current smokers.^25^

The cost of TDTS implementation was calculated for year 1 when program start-up costs were absorbed and for subsequent years. Year 1 TDTS costs were calculated as the sum of fixed and recurring costs in year 1. Total TDTS cost per smoker was calculated as the total program cost in year 1 divided by the number of smokers eligible to receive TDTS that year.^25^ These analyses were repeated to calculate the total TDTS cost in subsequent years. MUSC costs were incurred in 2015 and adjusted to 2017 dollar values based on the US Department of Labor CPI Inflation Calculator.^29^

### Potential Confounder Variables

Demographic and clinical covariates included in the cost models were patient age in years, race/ethnicity (white, black, Hispanic, other), insurance status (uninsured, Medicare, Medicaid, private, other), Charlson Comorbidity Index Score^30^ categories (none, mild, moderate, severe) and number of comorbidities.

### Statistical Analyses

To reduce potential TDTS exposure selection bias from nonrandomized data, inverse probability of treatment weighted propensity score methods^31^ were used to balance baseline characteristics on age, sex, race/ethnicity, insurance status, Charlson score, indicator variable for LOS (dichotomized as lower or higher than median), and comorbidities (eg, heart failure, chronic obstructive pulmonary disease, diabetes, hepatitis). Charlson Index Scores were converted to risk categories to fit the structure of non-normally distributed comorbidity data, following methods used by other researchers.^32^,^33^ Following propensity score weighting, baseline variables were reassessed to ensure similar distribution across baseline characteristics.

To test the hypothesis that exposure to the TDTS would reduce overall 1-year health care charges, we first compared actual health care charges for patients who did and did not receive the TDTS using student *t* tests. Total charges were calculated as the sum of inpatient, ambulatory surgery and ED charges. After standardizing inpatient data by DRG-group, we compared overall standardized inpatient hospital, ambulatory surgery and ED charges for patients with and without TDTS exposure. As a final step, we compared overall adjusted standardized total inpatient, ambulatory surgery and ED charges for patients with and without TDTS exposure. These analyses were repeated to evaluate cost outcomes by level of TDTS received. Because of the exploratory nature of the study, statistical corrections were not made for multiple comparisons.

Continuous and categorical variables were compared using *t* tests and χ^2^ tests, respectively. We then adjusted standardized inpatient, ambulatory surgery and ED charges for putative covariates including age, race/ethnicity, insurance status, Charlson Score and number of comorbidities in generalized linear models with a gamma distribution and log link. Covariates were added to the model to examine whether program exposure was statistically associated with standardized inpatient, ambulatory surgery and ED charges, after controlling for potential covariates. Marginal effects of TDTS exposure and TDTS intensity were estimated post regression. Statistical significance was assessed at the 0.05 α level, using STATA 15 (College Station, Texas).

## RESULTS

A cohort of 3081 current smokers with eligible index hospital admissions were evaluated, of whom 1441 were not exposed to TDTS and 1640 were exposed to some level of TDTS (764 to low intensity; 876 to high intensity). Over half of smokers were male (nonexposed=59.1%; exposed=52.5%), with a mean age of 47.6 and 49.4 years in the nonexposed and exposed groups, respectively. Mean LOS was 5.3 days in the nonexposed group and 5.0 days in the exposed group. As presented in Table 1, statistically significant differences for baseline characteristics including age, sex, insurance status, Charlson Score, and total comorbidities were observed between the nonexposed and exposed groups. However, after applying inverse probability of treatment weights, none of these differences remained statistically significant, demonstrating baseline characteristics were successfully balanced between exposure groups using propensity weighting methods.

**TABLE 1:**
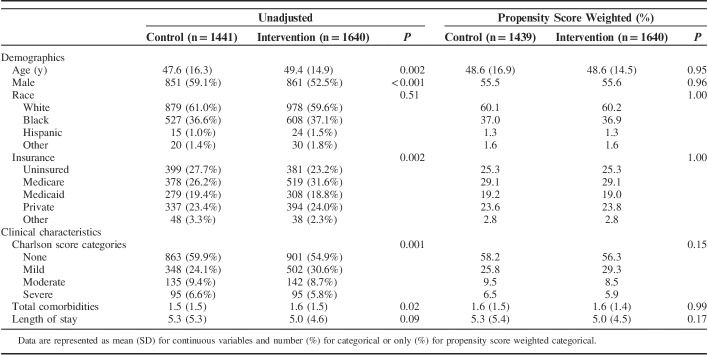
Characteristics of the Study Sample

Table 2 presents the association between TDTS exposure and 1-year health care charges. The overall unadjusted 1-year mean charge of care for TDTS exposed and unexposed patients were $52,539 (SD=$90,031) and $59,132 (SD=$105,283), respectively (*P*=0.03), favoring lower charges for patients in the TDTS exposed group. These overall charges were comprised of inpatient, ambulatory surgery and ED charges, each of which were in the direction of lower charges in the TDTS exposed group.

**TABLE 2:**
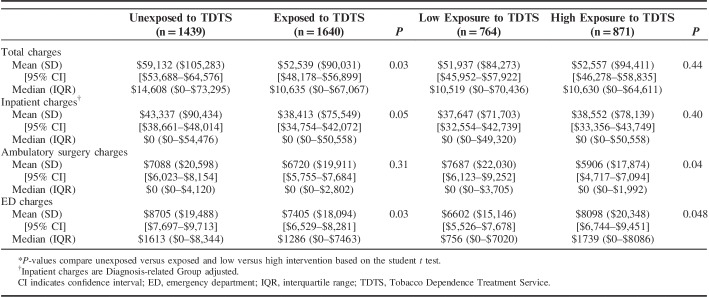
Unadjusted 1-Year Mean, Median, IQR, and CIs of Charges by Level of Exposure to the TDTS*

Overall unadjusted mean charges between the low and high intensity TDTS exposed groups were similar. Overall 1-year charges for the low versus high intensity groups were $51,937 (SD=$84,273) and $52,557 (SD=$94,411), respectively (*P*=0.44). In terms of the inpatient, ambulatory surgery and ED charges that contribute to the overall charges, the low and high intensity groups had similar inpatient charges. The high intensity group had higher ambulatory surgery and ED visit charges (*P*=0.036 and 0.048, respectively) compared with the low intensity group.

Table 3 presents the association between TDTS exposure and 1-year health care charges, controlling for covariates of age, race/ethnicity, insurance status, Charlson score, and number of comorbidities. Comparing overall health care charges for the TDTS exposed versus unexposed patient groups, mean charges for the TDTS exposed group were $7299 lower than for the unexposed group (*P*=0.047). Charges for inpatient, ambulatory surgery, and ED visits were $5242 (*P*=0.10), $699 (*P*=0.36) and $1547 (*P*=0.02) lower, respectively, for the TDTS exposed group compared with the unexposed group. Overall mean charges for the low intensity TDTS group versus the unexposed group also indicated lower charges in the low intensity group, but this result was not statistically significant at the *P*<0.05 level ($8006 lower, *P*=0.08). Overall mean charges for the high intensity TDTS group versus the unexposed group revealed a marginally lower charge of $6949 (*P*=0.12). Overall mean charges for the high versus low intensity groups were similar, with charges in the high intensity group on average $120 higher than in the low intensity group (*P*=0.98).

**TABLE 3:**
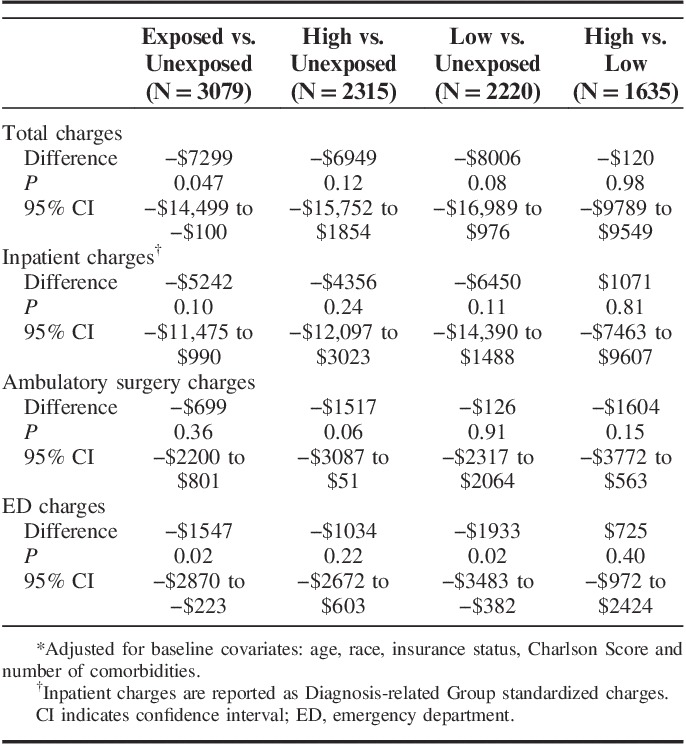
Adjusted Difference in 1-Year Health Care Charges by Level of Exposure to the Tobacco Dependence Treatment Service*

An overview of the costs for development and implementation of the MUSC Quits TDTS is presented in Table 4. The total TDTS cost in the first year of operation, which included program start-up costs, was estimated to be $158,140, which translates to $34.21 per smoker eligible for the service over a 12-month period. Removing start up costs, we estimate that the overall annual TDTS program cost would be $143,140, which translates to $30.97 per smoker eligible for the service over a 12-month period. TDTS costs were primarily driven by 2 factors: (1) staffing costs for the TTS and program manager which accounted for 52% of the overall costs; and (2) the TelASK cost that is charged per estimated number of hospitalized smokers, which reflected 45% of overall costs.

**TABLE 4:**
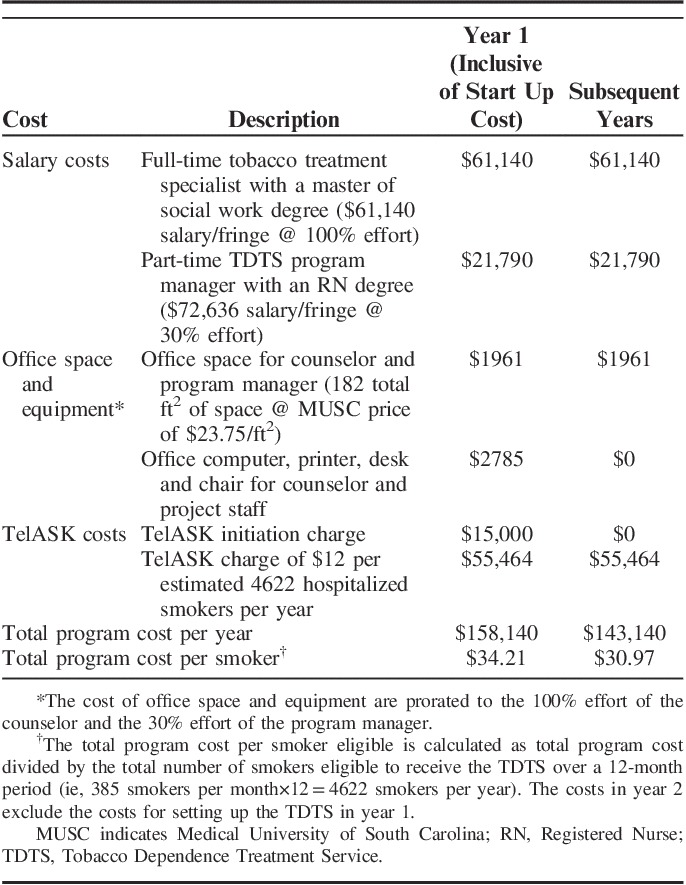
Cost of Implementing the TDTS

## DISCUSSION

The primary findings were that health care charges were $7299 lower among hospitalized smokers exposed to the TDTS (*P*=0.047). In SC, the cost to charge ratio is between 30% and 40%, meaning actual health care costs are ∼30%–40% of charges, resulting in an average of $2190–$2920 lower cost per smoker who received the TDTS service. Within the context of our study in which 1640 patients received the TDTS over an 8-month period, this would translate into a health care cost savings ranging from $3.6 to $4.8 million, taking into account the cost of program delivery and cost savings per patient. These data suggest that between 54 and 72 smokers would need to receive TDTS services to cover the cost of delivering the service. The overall costs of implementing the TDTS were modest relative to the potential savings in estimated health care costs.

The cost for delivering the TDTS compares favorably with the cost of programs relying on either clinical staff or IVR technology to deliver follow-up cessation support calls. Typical program costs for provision of inpatient smoking cessation services and follow-up reported in the literature range from $74 to $189 per patient.^19^,^20^,^23^ The lower average program cost per smoker with the MUSC TDTS likely reflects the minimal nature of the intervention delivered which involved one full-time TTS, a part-time TDTS program manager, and provision of automated IVR follow-up calls. Because of limited staffing and budget, the MUSC TDTS only reached 53% of the eligible smoker population in the hospital and did not include provision of medications to patients. A larger investment in the service would have allowed us to reach more smokers and in turn might further reduce health care costs, although we did not observe differences in health care charges between low and high intensity arms of the TDTS.

This study contributes most notably to the literature evaluating possible benefits of providing smoking cessation services to hospitalized patients by using actual program and health care utilization data, rather than modeling these charges as other studies have done. In this study we utilized actual TDTS costs, along with actual health care charges for inpatient, ambulatory surgery and ED charges accrued by patients within 1-year after hospital discharge. While these data are observational, the findings show that a TDTS consistent with JC standards for smoking cessation can be affordably implemented and potentially yield substantial health care savings.

Limitations should be considered in interpreting study findings. First, we did not have a true control group of patients unexposed to the TDTS. Instead we constituted a comparison group of patients who were eligible for the service but did not see the TTS and did not respond to IVR follow-up calls after hospital discharge. Differences in characteristics of patient groups exposed and unexposed to the TDTS could account for downstream differences in health care charges. For example, it is possible some patients did not receive bedside counseling or respond to follow-up calls because they were very healthy (eg, discharged rapidly) or very ill (eg, too ill to speak with TTS). To reduce potential program exposure selection bias, propensity weighting was employed to balance baseline patient characteristics, along with covariate adjustment in our statistical models. The results of adjusted and unadjusted analyses were similar, suggesting our findings are robust. We acknowledge that an randomized controlled trial design with hospitals assigned to JC-styled TDTS versus usual care would provide a more rigorous test of impact of the TDTS on health care charges. We believe the evidence from this single institution study warrants a more rigorously designed prospective follow-up study to determine if these findings can be replicated. Second, this study was not powered to detect differences between subgroups with low, high and no exposure to the TDTS. Despite limited sample size, patients exposed to the TDTS had on average $7299 lower health care charges over a 1-year follow-up period compared with patients not exposed to the TDTS. Third, we were unable to obtain actual cost data, therefore, charges were utilized as proxy for health care costs. Hospital inpatient charges for the same type of admission can vary widely based on hospital mission (for-profit, nonprofit, etc.) and insurance status of the individual. To reduce this variability in inpatient charges, we calculated standardized charges by DRG by summing all admission charges for each DRG and dividing by the number of admissions to obtain a mean charge for each DRG. The mean charge per DRG was then applied to each admission based on its assigned DRG. The use of standardized DRGs has potential to inflate *P-*values.

To date, tobacco cessation has not been established as an influential driver of health care cost reduction. This study provides evidence that a TDTS consistent with JC smoking cessation standards may help to markedly reduce health care charges in those identified as smokers upon hospital admission over a 1-year period consistent with what might be expected given the well documented hazards of smoking. While these findings need replication in other health care institutions to confirm the magnitude of observed benefit, the results should encourage health care administrators to consider investing in a JC-styled TDTS.
